# Using patients’ observations to evaluate healthcare workers’ alcohol-based hand rub with Pulpe’friction audits: a promising approach?

**DOI:** 10.3205/dgkh000455

**Published:** 2023-11-29

**Authors:** Fanny Velardo, Muriel Péfau, Raymond Nasso, Pierre Parneix, Anne-Gaëlle Venier

**Affiliations:** 1 Center for Prevention of Healthcare Associated Infections of Nouvelle Aquitaine, CPias Nouvelle-Aquitaine, Bordeaux, France; 2 Center for Prevention of Healthcare Associated Infections of Guadeloupe, CPias Iles de Guadeloupe, Pointe-à-Pitre, France

**Keywords:** evaluation, hand disinfection, patient observations, alcohol-based hand rub, prevention infection

## Abstract

**Background:**

Hand hygiene plays an important role in the transmission of nosocomial infections from healthcare workers (HCW) to patients. Patients could play a key role in improving hand hygiene by sharing their experience of the HCW’s practices. Already in 2019, the French national mission of transversal support for actions to prevent healthcare-associated infections proposed the national “Pulpe'friction” audit, to assess HCW’s reported practices, social representations, and barriers to using alcohol-based hand rubs (ABHR). This audit consisted of a positive discussion between an auditor and the HCW as well as patients, which led the HCW to declare their real practices and the barriers they faced in the field and the patients to report about the HCW’s ABHR practices and the information they received about when they should perform hand hygiene

**Objective:**

To assess whether an association existed between HCW’s reported ABHR compliance and patients’ declarations about HCW’s compliance in the Pulpe’friction audit data.

**Methods:**

Data from Pulpe’friction were collected from 1^st^ January to 31^st^ December 2019, before the COVID-19 pandemic. Mixed linear models were performed to analyze the association between self-reporting by HCW and patients, regarding hand rubs performed by HCW prior to patient care.

**Results:**

There was a positive association between patients’ observations and HCW’s declared practices regarding the frequency of with which professionals performed hand rubs before patient contact. This indicates that professional and patient statements show the same tendency. The positive association was found in hospitals for patients under 45 and over 64 years old and for paramedics, but not for physicians and not in nursing homes or long-term care facilities. Patients felt more motivated to observe and evaluate HCWs’ practices if they had received information about how to correctly wash their hands.

**Conclusion:**

Patients agreed to be involved in the evaluation or professional practices. The patients’ observations were positively associated with HCWs reports. New indicators taking patients’ observations into account could be interesting.

## Zusammenfassung

**Hintergrund:**

Die Händehygiene spielt eine wichtige Rolle bei der Übertragung nosokomialer Erreger vom Gesundheitspersonal auf Patienten. Patienten könnten eine Schlüsselrolle bei der Verbesserung der Händehygiene spielen, indem sie ihre Erfahrungen mit den Gesundheitspersonal teilen. Bereits im Jahr 2019 schlug die französische nationale Mission zur transversalen Unterstützung von Maßnahmen zur Prävention Health Care assoziierter Infektionen (HAI) das nationale „Pulpe'friction“-Audit vor, um die vom Gesundheitspersonal gemeldeten Praktiken, Darstellungen und Hindernisse für die Alkohol basierte Händedesinfektion zu bewerten. Das Audit beinhaltete eine positive Diskussion zwischen dem Auditor, dem Gesundheitspersonal und Patienten, die dazu führte, dass die Gesundheitspersonal dazu veranlasste, die tatsächlichen Praktiken und Hindernisse, mit denen es vor Ort konfrontiert war, darzulegen, und das die Patienten dazu veranlasste, über die Praktiken der Händedesinfektion des Gesundheitspersonals und die Informationen, die sie über ihre eigene Handhygiene erhielten, zu berichten.

**Zielsetzung:**

Es sollte beurteilt werden, ob ein Zusammenhang zwischen der vom Gesundheitspersonal berichteten Compliance der Händedesinfektion und der von Patienten berichteten Compliance des Gesundheitspersonal der Händedesinfektion in den Pulpe’friction-Auditdaten besteht.

**Methoden:**

Die Daten von Pulpe’friction wurden vom 1. Januar bis 31. Dezember 2019, also vor der COVID-19-Pandemie, erhoben. Mit Hilfe gemischter linearer Modelle wurde der Zusammenhang zwischen der Selbstauskunft des Gesundheitspersonals zur Einhaltung der Compliance der Händedesinfektion und der Einschätzung der Patienten hinsichtlich Einhaltung der Compliance durch das Gesundheitspersonal vor der Behandlung analysiert.

**Ergebnisse:**

Es bestand ein positiver Zusammenhang zwischen der von Patienten und der vom Gesundheitspersonal eingeschätzten Praxis hinsichtlich der Häufigkeit der Durchführung der Händedesinfektion vor der Behandlung, was bedeutet, dass die Aussagen der Fachkräfte und der Patienten in die gleiche Richtung gehen. Dieser Zusammenhang wurde in Krankenhäusern bei Patienten unter 45 Jahren und über 64 Jahren und bei nichtärztlichen Berufsgruppen im Bereich des Rettungsdienstes, nicht aber bei Ärzten und nicht in Pflegeeinrichtungen festgestellt. Die Patienten fühlten sich stärker an der Beobachtung und Bewertung der Praktiken der Gesundheitsdienstleister einbezogen, wenn sie Informationen darüber erhalten hatten, wann und wie die Hände zu desinfizieren sind.

**Fazit:**

Die Einschätzung der Patienten stand in Übereinstimmung mit den Erklärungen des Gesundheitspersonals zur Compliance der Händedesinfektion. Neue Indikatoren, die die Erfahrungen der Patienten einbeziehen, könnten interessant sein, und die Patienten erklärten sich bereit, in die Bewertung der beruflichen Praxis einbezogen zu werden.

## Introduction

Hand hygiene plays an important role in interrupting the transmission of nosocomial infections from healthcare workers (HCW) to patients [1], [2], [3], [4], [5], [6], [7]. There are two ways of performing hand hygiene: rubbing hands with an alcohol-based hand rub (ABHR) or washing them with soap and water. When hands are visually clean and dry, ABHR is preferable, as it is microbiologically more effective [2], [4], [5], [7], better tolerated by the skin [2], [8], [9], more user-friendly, does not need towels for drying because it is not dependent on water outlets, has a shorter exposure time (15 sec) [10], [11], [12], [13], is more ecological [14] and is associated with higher compliance of implementation [7]. 

Patients can play an important role in hand hygiene improvement: first by correctly disinfecting their hands and second by sharing their observations of what they see in a healthcare setting. The idea of involving healthcare recipients in the design and implementation of health policies was born in the mid-1990s in Canada and became increasingly important all over the world. Patients’ feedback provides a unique point of view to improve health care pathways and guide HCW and decision-makers. French healthcare recipients are nowadays involved in all decision-making authorities, from individual care to boards of directors, with a power of decision. Patients’ observations remain a largely neglected method in evaluating HCWs’ ABHR practices. There is still no information about the accuracy of patients’ observations, and thus whether or not interest even exists in involving them in the evaluation of HCWs’ hand hygiene practices. In 2018, the Ministry of Health and the French National Public Health Agency Santé Publique France created the French national mission of transversal support for actions to prevent healthcare-associated infections (called “MATIS”) to facilitate the prevention of HAIs. 

In 2019, MATIS proposed the national “Pulpe'friction” audit to assess HCWs’ reported hand-rub practices and barriers to using ABHRs (method in [15]). This audit was developed in line with WHO guidelines to monitor the use of hand disinfectants by medical staff and to promote improvement measures consists in a positive discussion between an auditor and each HCW and patient, inducing the HCW to report their real practices as well as the barriers they faced in the field, in addition to encouraging the patients to share the HCW practices they saw and whether they received information about the moments at which they could, as a patient, perform a hand disinfection. This audit can be performed each year, providing the infection control team with useful data for adapting/modifying their actions accordingly.

This study determined whether there was an association between the HCWs’ reported practices and the patients’ observations of HCWs’ practices. 

## Materials and methods

Pulpe’friction audit began in 2019 and is still ongoing. It is one of the official tools of the French National Hand Hygiene Day. Its methodology follows WHO guidelines and the French Hygiene Society (SF2H). Its name is inspired by the fingertips (“pulpe des doigts” in French), an important part during hand rubbing, and by ABHR, called “friction” in French. The Pulpe’friction quick audit is based on a partnership between the person interviewed and the interviewer, who collects reported practices through a short individual questionnaire of less than 10 questions, lasting 5 to 10 minutes. The methodology provides language elements to lead the HCW report practices that correspond as exactly as possible to HCW’s real practices. HCWs were asked how frequently they performed ABHR in four situations: “before patient contact”, “before an invasive technique”, “after patient contact”, “after contact with patient surroundings”, using a scale from 0 (=never) to 10 (=always). 

Patients were asked about their age, how often they saw HCWs performing hand rubs before touching them in the past few days (0=never to 10=always), the importance they gave to this action of hand disinfection (0=not important at all to 10=absolutely necessary), if they think that patients should help evaluating professionals’ hand rubbing (yes or no), if they received any information during their stay about when they should perform hand hygiene (yes or no), and how important is it for them to receive that kind of information (0=not important at all to 10=absolutely necessary). To be interviewed, patients had to be conscious and able to understand questions. The data were entered online on the Pulpe’friction web app on the Healthcare Associated Infection Prevention Network (RéPias) website (www.preventioninfection.fr). Infection control teams obtain automated results with personalized advice for each session in order to help to choose adapted actions.

### Ethics approval and consent to participate

Data were anonymized, and participants consented to their use. The database management was approved by the ethics committee of the Guadeloupe University Hospital (reference number A11-20-02-21-BOX-IMPACT1), in accordance with the General Data Protection Regulation of the EU, the French National Commission for Data Protection (CNIL) and French regulations. 

### Study population

The study included the data of the hospitals (public hospitals and clinics), nursing homes and establishments for disabled adults which participated in the audit from the1^st^ January 2019 to the 31^st^ December 2019. HCWs were grouped into two categories: physicians and paramedics (such as nurses, auxiliary nurses, physiotherapists). 

### Statistical analysis

It could be considered that the data reported by the patients in a same ward were not independent (cluster effect, which could also considered for the HCWs in the same ward). So, data were pooled to obtain average scores per ward. Mixed generalized linear models were performed to assess if there was an association between the average patients’ declaration and the average HCWs’ declaration regarding hand rubbing before patient contact. Therefore, at least two patients and two HCWs per ward were needed to calculate means and perform regression models. A subset of 148 facilities met the criteria, and the analysis of the non-selected facilities showed that they were not different from the selected ones in terms of specialty, profession distribution and patient characteristics. Generalized linear models included a random effect of the facility to consider the correlation of practices within a facility. Multivariate models were performed using significant variables and relevant parameters according to the literature (age of patients, professional occupation, ward specialty). A weighting was implemented for the number of people audited within wards. R studio^©^ version 1.2.5033 [16] was used to perform the statistical analysis. A p-value <0.05 was considered significant. 

## Results

Overall, 16,285 HCW and 5,299 patients answered from January 1^st to^ December 31^st^, 2019. Respondents came from 307 healthcare facilities, including 274 hospitals and 33 nursing homes from 16 of the 17 French regions. There were 5,247 patients and 15,761 HCWs in hospitals, and 52 patients and 524 HCWs in nursing homes (Table 1 (Tab. 1)).

HCWs declared an average compliance of 94% (9.4/10) for the situation “before the insertion of an invasive device” and assigned an importance of 97% to this situation. They reported an average compliance of 84% for the situation “after touching the patient” and attached an importance of 92% to this situation. Two situations were below 80%: “before touching the patient” (compliance 71%, importance given 87%) and “after touching the patient's immediate environment” (compliance 76%, importance given 85%). Around 72% of patients agreed to participate in the evaluation of professional practices, including 72.6% in hospitals and 51.9% in nursing homes. The desire to become involved varied across wards, with 54.2% in emergency wards compared to 75% in surgical wards. Around 36% of patients reported that they received information about when to perform hand hygiene and attached an importance of 80% to receiving this knowledge. Patients hospitalized for hemodialysis were the most informed (74%), while patients in laboratories and medical imaging were the least informed (10%). Patients observed that professionals had a compliance rate of 76% for the situation “before a contact for a care” (and attached an importance of 90% to this situation. Patients were 6.7 times more likely to be informed if they were hospitalized in a hemodialysis sector, 38% more likely in psychiatry, follow-up and rehabilitation care, or in long-term care, but 73% less likely to be informed if they were in technical sectors than in infectious diseases wards. They were 11.5% less likely to be informed if aged ≥65 years (Table 2 (Tab. 2)). 

A subset of 148 facilities was used to perform regression models between patients’ and professionals’ answers (140 hospitals and 8 nursing homes) for a total of 10,715 respondents (4,751 patients and 5,964 HCWs). These results were expressed as percentages, which implies multiplying the coefficients and confidence intervals displayed in Table 3 (Tab. 3) by 10, to facilitate understanding and interpretation. There was a positive association between the observations by patients and professionals’ report regarding the frequency of professional hand rubbing: here, a β of 0.13 means that the patient frequency of observation increased by 1.3% when the professional report increased by 10% (CI95%=[0.01–0.02], p=0.001). The association was found in hospitals (IC95%=[0.01–0.02], p=0.001) but not in nursing homes (IC95%=[–0.36–0.31], p=0.85), for ≤44 years old (IC95%=[0.02–0.06], p<0.001) and ≥64 years old (IC95%=[–0.01–0.02], p=0.84) but not 45–64 years old (IC95%=[–0.01–0.02], p=0.84) (Table 3 ). In parallel, the association was found for paramedics (IC95%=[0.01–0.02], p=0.03) but not for physicians (IC95%=[–0.01–0.01], p=0.36). 

The average frequency of patient’s observation of professional hand rubbing increased by 4% when the importance attributed to this practice increased by 10% (CI95%=[0.36; 0.44], p<0.001). The average frequency of patient’s observation of professional hand rubbing increased by 8.5% when patients had received prior information about hand hygiene CI95%=[0.7; 1], p<0.001. It decreased by 5.1% when they were ≥65 years old (CI95%=[–0.68; –0.36], p<0.001) (Table 4 (Tab. 4)). 

Also, the importance attached to professional hand rubbing increased by 1.7% (95% CI=[0.04; 0.27], p=0.001) and the desire to become involved in the evaluation of professional practices by 37% (odds ratio=1.37, CI95%=[1.20; 1.55], p<10^–3^) when patients had received prior information about when they should perform hand hygiene.

## Discussion

These results suggested a clear association between HCWs’ declared practices and patients’ observations. These results seem important since patient perception of hand rubbing remains mostly unknown. These results suggested that patients’ observations could be integrated into a new quality- and safety-of-care indicator, a suggestion which has already been put forth in the literature [17].

This study has some limitations. The sample was large enough to be considered representative of French hospitals, but not for nursing homes. The questions were designed to limit interpretation bias and facilitate understanding and honesty of the responses. The Hawthorne effect was also avoided, but the bias of social desirability cannot be excluded with this method. 

There was a strong association between the perceptions of the patients under 45 or over 64 and professional’s declarations. In contrast, this association was not significant for 45–64 year-old patients. This curvilinear relationship is known in psychology as the Erikson’s psychosocial theory, saying that “The individual progresses through a number of stages or crises”. Anxiety about death varies with age, with a peak of anxiety when people are between 45–64 years old [18]. Adults aged 45–64 could potentially be more anxious about their death, and less likely to check on HCWs’ practices, possibly as a result of being more prone to shock and denial. 

No association was found between nursing home residents’ observations and professionals’ self-reports. The literature has shown that nursing home residents value the human relationships over other needs [19]. The importance of follow-up is centered on medical and social support, and technical care is less frequent than in the other wards. However, further investigations should be made in the future with a larger sample and variety of health and social welfare institutions.

In France, medical school deans expressed the will to engage patients as teachers in university programs [20]. Patients could become mentors by creating a space for reflexion and express what they experience as recipients in the healthcare system [12]. The French hospital certification now requires audits that include patient feedback through satisfaction scores via different indicators. 

Patients’ observations are collected in Pulpe’friction to make HCWs aware of the experiences and opinions of the people to whom they provide care. It helps some practices make more sense to HCWs. This study confirmed the willingness of the patients to be involved, as two-thirds of them agreed to evaluate professional ABHR. As shown by the results of the Patient Reported Outcomes (PROMs) and Patient Reported Experience (PREMs) collection methods, collecting patients’ experiences/observations is informative and provides feedback to healthcare teams, particularly in surgery [21], [22], [23] and neurology [24]. PREMs collect information on how patients experience their care, through satisfaction measurements and subjective-experience questionnaires. The feedback from patients using PREMs methods can enable HCWs to modify their practices. 

To collect patient’s experiences/observations also allows the patients to become actors in the healthcare organization. It is now clear that representation of hand hygiene should be improved, whether inside or outside the hospital, as patient awareness regarding hygiene practices can help to reduce the prevalence of HAI [25], [26]. Participation in the evaluation of professional practices could also include the patient’s family, as it seems to improve the quality and safety of care [27].

## Conclusions

This study showed that patients’ reports are correlated with healthcare professionals’ declared practices. Further tools could be developed based on Pulpe’friction audits to assess other practices in a social and human approach, including patients and their family. 

## Notes

### Competing interests

The authors declare that they have no competing interests.

All authors are active at the French national mission of transversal support for actions to prevent healthcare-associated infections (Mission d’Appui Transversal de prévention des Infections associées aux Soins – MATIS)

### Funding source

None

### Consent for publication

All authors reviewed and approved the publication of this manuscript

### Acknowledgments

Authors thanks the infection control teams, HCWs and patients who participated to the Pulpe’friction quick audits in 2019.

### Author’s ORCID

The ORCID ID of Venier AG is: 0000-0002-5077-9820

## Figures and Tables

**Table 1:**
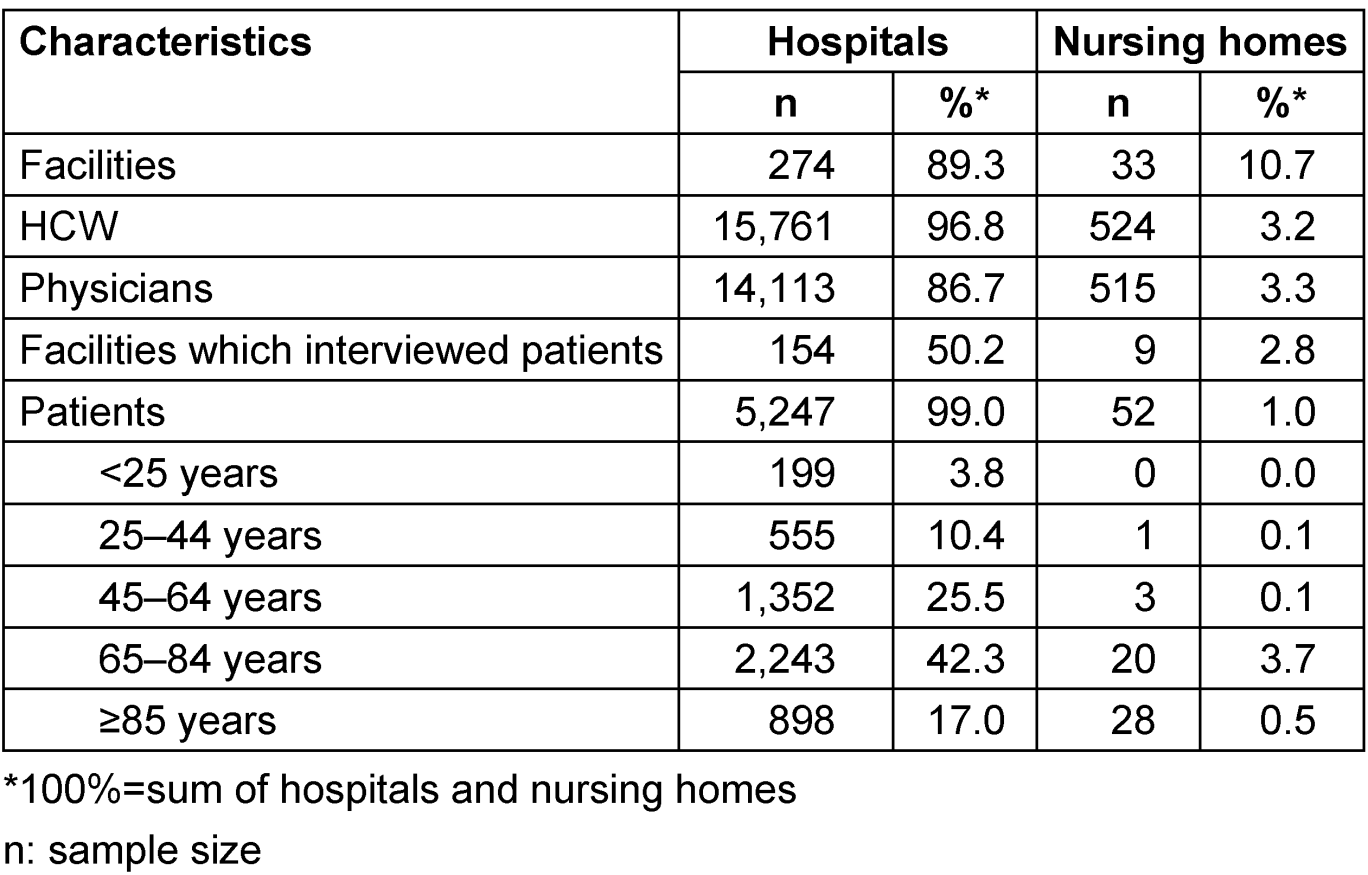
Characteristics of the study population

**Table 2:**
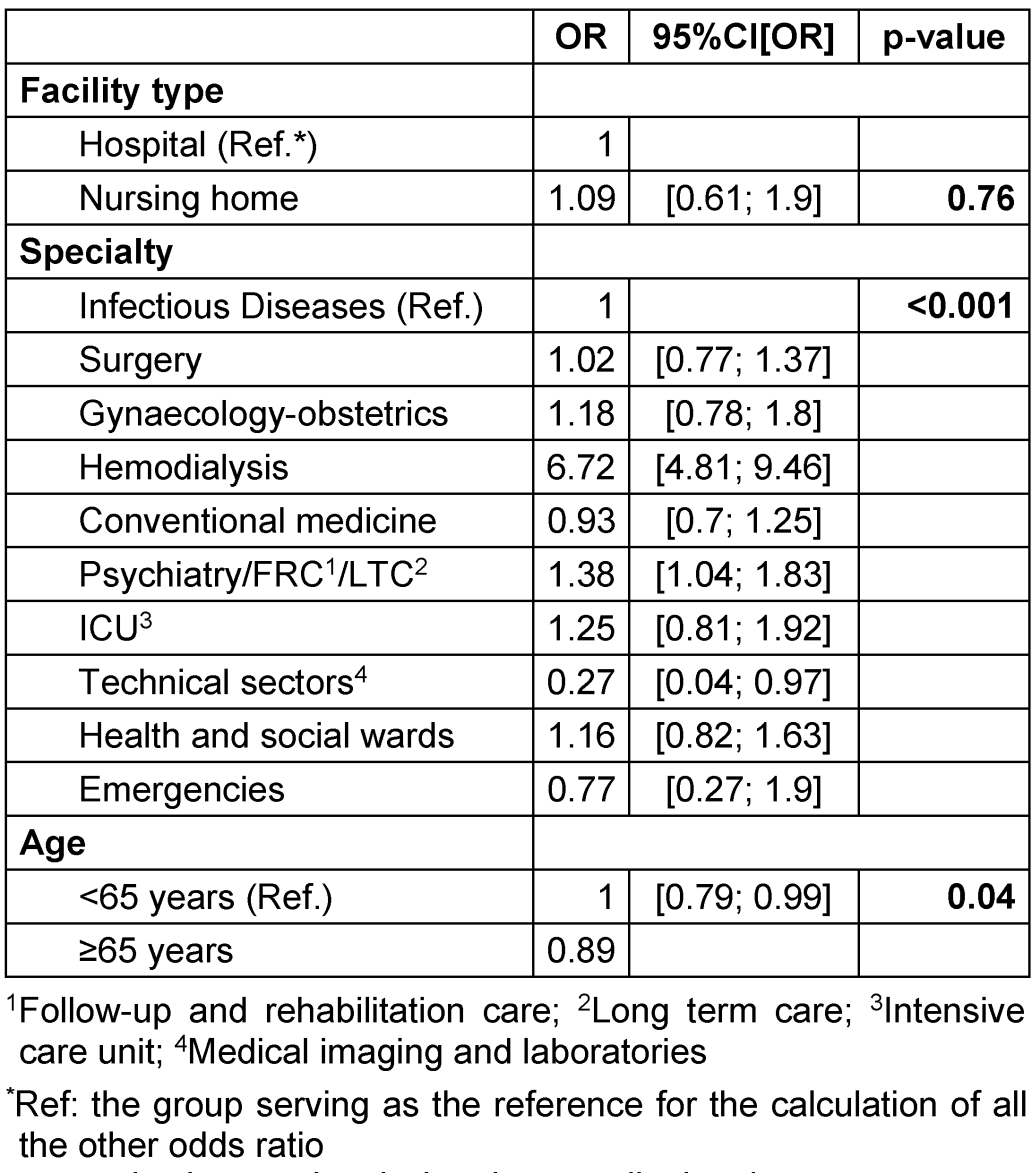
Mixed generalized models regarding patient’s information level about hand hygiene

**Table 3:**
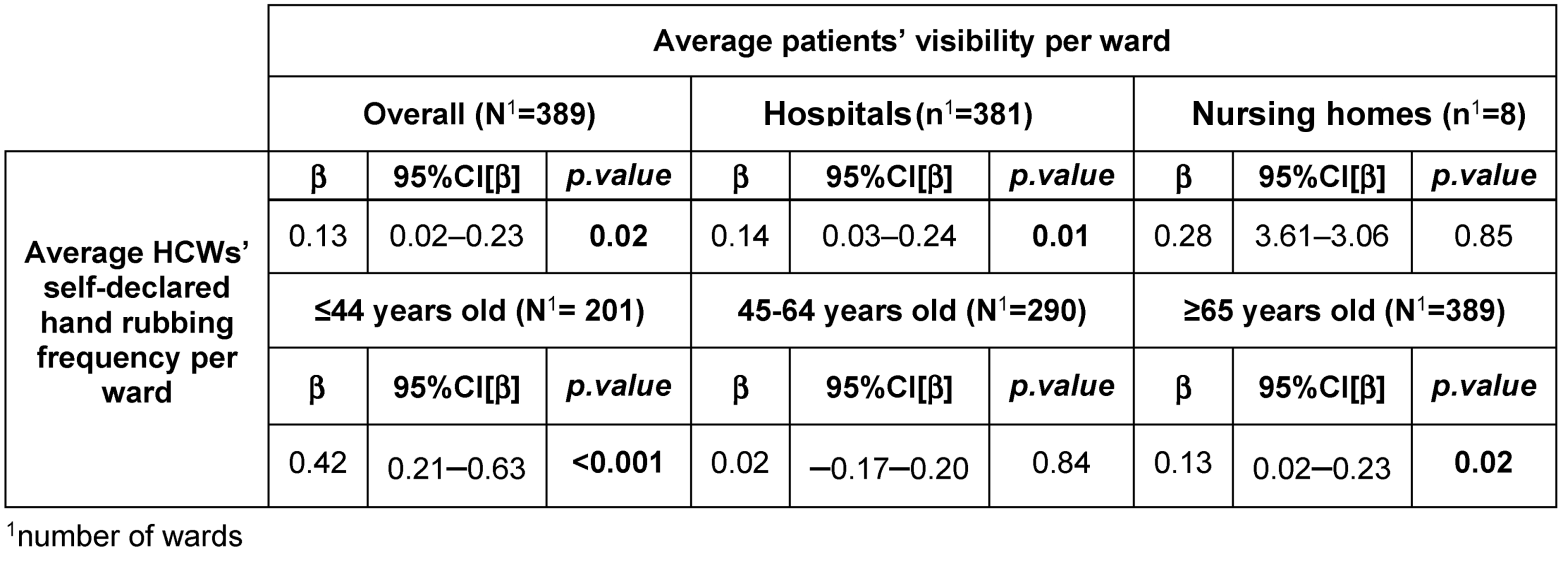
Mixed generalized linear models of average patients’ observation of professional hand rubbing before care according to average healthcare professionals’ self-declared practices

**Table 4:**
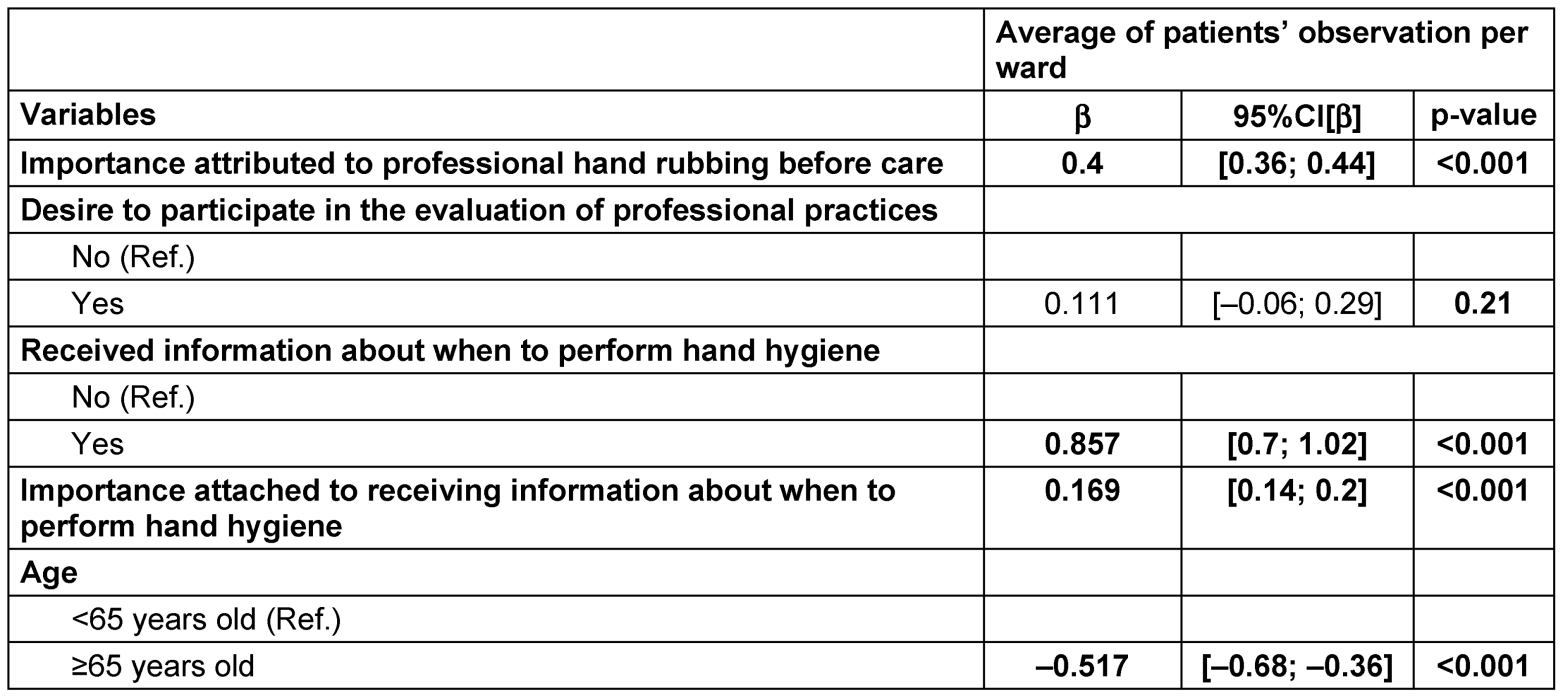
Mixed generalized models of average patients’ observations of professional hand rubbing before care
